# Tensile Retention of Lithium Disilicate and Zirconia Crowns Cemented to One-Piece Zirconia Implants: A Pilot In Vitro Study of Cementation Protocol, Resin Cement, and Micro-CT Cement Morphology

**DOI:** 10.3390/ma19163518

**Published:** 2026-08-19

**Authors:** Veranda Azizi Bunjaku, Ying Xue, Blerina Azizi Veseli, Nenad Drvar, Ivica Pelivan

**Affiliations:** 1 School of Dental Medicine, University of Zagreb, HR-10000 Zagreb, Croatia; vazizi@sfzg.unizg.hr (V.A.B.); baveseli@sfzg.unizg.hr (B.A.V.); 2 Department of Clinical Dentistry, UiT The Arctic University of Norway, 9037 Tromsø, Norway; ying.xue@uit.no; 3 Topomatika d.o.o., HR-10000 Zagreb, Croatia; n.drvar@topomatika.hr

**Keywords:** zirconia implants, lithium disilicate, resin cement, tensile retention, micro-CT, cement thickness, implant prosthodontics

## Abstract

**Highlights:**

- In this pilot dataset, crown material was associated with differences in the maximum recorded force at first failure (median 596.05 N for zirconia versus 347.20 N for lithium disilicate; exact Mann–Whitney *p* = 0.017).
- The AH protocol showed higher observed maximum forces than the A-AH protocol (medians 614.38 versus 304.24 N; *p* < 0.001).
- No statistically significant association between resin cement type and the maximum recorded force was detected in this pilot dataset (*p* = 0.210); with four specimens per subgroup, this is an absence of evidence rather than evidence of no association.
- Zirconia crowns showed higher observed pull-out loads than lithium disilicate crowns in this pilot dataset; failure occurred by crown debonding in 27 of 32 specimens, by crown fracture in 4 and by implant fracture in 1.
- The preliminary findings suggest that the cementation protocol may warrant greater consideration than the cement type.
- In the companion micro-CT investigation of the same specimens, the protocol associated with higher observed retention was also associated with more excess cement; excess cement was not measured in the present analysis.
- In the lithium disilicate specimens only, cement thickness varied over a total range of 1.67 µm (253.29–254.96 µm); an unadjusted univariable association with debonding was observed on that scale, but the range is confounded with cementation protocol and no clinically meaningful or independent morphology effect is demonstrated. No micro-CT data exist for the zirconia specimens.
- Cementation strategies should balance potential retention benefits against biological risk; these preliminary findings require confirmation.

**Abstract:**

This pilot in vitro study explored the tensile retention of lithium disilicate and monolithic zirconia crowns cemented onto zirconia one-piece implants using two resin cements and two cementation protocols. In addition, the relationship between micro-computed tomography (micro-CT)-derived cement layer characteristics and retention was explored for lithium disilicate crowns. Thirty-two implant–crown assemblies were prepared using 16 lithium disilicate and 16 zirconia crowns. Specimens were cemented with either an adhesive resin cement (Panavia V5) or a self-adhesive resin cement (SpeedCem Plus) using two protocols: conventional apical-half cementation (AH) and an abutment-assisted apical-half protocol (A-AH). Cement thickness and porosity for lithium disilicate crowns were obtained from a previously published micro-CT analysis of the same specimens; no micro-CT measurements were available for the zirconia specimens. Tensile pull-out testing was performed using a universal testing machine. The primary outcome was the maximum recorded force at the first observed mechanical failure, irrespective of the mode of that failure, so that all 32 specimens contributed a value. Failure occurred by crown debonding in 27 specimens, by crown fracture in 4 and by implant fracture in 1. For the primary outcome, the maximum recorded force was lower for lithium disilicate than for zirconia crowns (medians 347.20 versus 596.05 N; exact Mann–Whitney *p* = 0.017) and lower with the A-AH than with the AH protocol (medians 304.24 versus 614.38 N; *p* < 0.001), whereas the difference between the two resin cements was not statistically significant (medians 438.88 versus 550.83 N; *p* = 0.210). The highest observed mean maximum load was recorded for zirconia crowns cemented with Panavia V5 using the AH protocol (729.9 ± 237.7 N), whereas the lowest observed mean maximum load was recorded for lithium disilicate crowns cemented with Panavia V5 using the A-AH protocol (219.7 ± 105.1 N). In a secondary, cause-specific exploratory analysis restricted to crown debonding (27 events, 5 specimens censored at fracture), Cox proportional hazards regression on the applied-force scale gave hazard ratios of 3.75 (95% CI 1.40–10.01) for lithium disilicate versus zirconia, 6.47 (2.39–17.53) for A-AH versus AH and 1.82 (0.76–4.39) for Panavia V5 versus SpeedCem Plus. For lithium disilicate crowns, exploratory factorial ANOVA indicated that cementation protocol was associated with differences in cement thickness (*p* = 0.035), while cement type was associated with differences in porosity (*p* < 0.001). All 16 lithium disilicate cement thickness observations lay between 253.29 and 254.96 µm, a total span of 1.67 µm. Within that extremely restricted range, a univariable exploratory Cox model expressed per 0.1 µm gave a hazard ratio for debonding of 1.24 (95% CI 1.03–1.48; *p* = 0.024); this is an unadjusted association across a range that is itself associated with cementation protocol, and it does not demonstrate a clinically meaningful or independent effect of cement thickness. No association was detected for total porosity (0.959 per percentage point, 0.717–1.283); that interval is wide and indicates absence of evidence rather than evidence of no association. Within the limitations of this pilot in vitro study—four specimens per subgroup, wide confidence intervals and no adjustment for multiplicity—the findings suggest that crown material and cementation protocol may be associated with retention patterns. They are exploratory and hypothesis-generating and require confirmation in larger, independently powered studies.

## 1. Introduction

### 1.1. Restorative Materials and Bonding Strategies in Ceramic Prosthodontics

Lithium disilicate glass-ceramics and yttria-stabilized zirconia (YSZ) are metal-free materials used in fixed prosthodontics owing to their favorable mechanical properties, esthetic properties, and biocompatibility [1,2]. Lithium disilicate exhibits high flexural strength, tooth-like translucency, and is commonly used in adhesively bonded ceramic crowns [1,3,4].

Bonding is achieved via hydrofluoric acid etching followed by silanization, enabling micromechanical and chemical adhesion to the resin cement [1,3,4]. Zirconia, most commonly a 3 mol% yttria-stabilized tetragonal zirconia polycrystal (3Y-TZP), is an oxide ceramic whose strength and fracture toughness are primarily attributed to transformation toughening [1,5]. Since it is not amenable to hydrofluoric acid etching, bonding strategies typically involve airborne-particle abrasion and phosphate-functional monomers, such as MDP, delivered via primers and/or resin cements [6,7].

In implant dentistry, zirconia is an alternative to titanium for abutments and fixtures owing to its tooth-colored appearance, favorable biocompatibility, and reduced plaque accumulation [8,9]. Preclinical and clinical studies have indicated that zirconia implants achieve osseointegration and short- to mid-term survival rates comparable to those of titanium implants [9,10].

### 1.2. One-Piece Zirconia Implants: Mechanical and Biological Considerations

One-piece zirconia implants integrate the endosseous component and transmucosal abutment into a single structure and have demonstrated acceptable survival and marginal bone stability in prospective cohorts and long-term case series [11,12,13].

Recent systematic reviews have reported promising short- to medium-term outcomes for zirconia dental implants. Gül et al. (2024) found that prospective studies of one-piece zirconia implants demonstrated 5-year survival rates of 95% to 98.4%, while Mohseni et al. (2023) reported a 10-year cumulative survival rate of 95.1% across zirconia implant studies [14,15]. However, individual long-term investigations have raised concerns, as Kohal et al. (2026) reported a 10-year cumulative survival rate of only 73.3% for a specific one-piece zirconia implant system, with significant peri-implant bone loss [16]. These findings highlight that while the overall evidence base is encouraging, long-term performance may vary substantially across implant designs, and further investigation of factors influencing the performance of zirconia implant restorations remains warranted.

However, the one-piece design precludes screw retention, requiring the crowns to be cemented directly onto the zirconia abutment [17,18].

Limited abutment customization and the frequent presence of submucosal crown margins can compromise visual control during cement removal, increasing the risk of residual excess cement. Residual cement is a local risk factor for peri-implant mucositis, peri-implantitis, and marginal bone loss, particularly when margins are positioned submucosally [19,20,21]. Cementation strategy is therefore critical for balancing mechanical retention and biological risk in implant-supported ceramic restorations.

### 1.3. Cementation and Retention of Implant-Supported Ceramic Restorations

In vitro studies on lithium disilicate or zirconia crowns cemented to zirconia abutments or titanium bases have reported higher retentive forces for adhesive and self-adhesive resin cements than for conventional or provisional cements, and have emphasized the role of surface pretreatment and MDP-containing systems [22,23,24,25]. For lithium disilicate crowns on zirconia custom abutments, Panavia 21 and a dedicated hybrid abutment resin cement provide higher retentive strength than temporary cements [3,22]. Comparable results have also been reported for zirconia- and polymer-based copings bonded to titanium bases, where resin-based cements are stronger and have higher fatigue resistance than conventional or provisional cements [24,26,27]. The influence of airborne-particle abrasion appears to be material- and substrate-dependent; on microgrooved titanium bases, it reduces the retention of zirconia restorations [26], whereas for polymer copings, it does not improve bonding unless a hybrid-abutment cement is employed [24]. Thin cement films have been linked to higher retention when resin cement is used [28].

Nevertheless, most studies have focused on screw-retained restorations supported by titanium bases or two-piece zirconia abutments, whereas data on crowns cemented onto one-piece zirconia implants are limited. Evidence regarding the influence of cement selection on the mechanical performance of restorations cemented directly onto one-piece zirconia implants is limited [8,9,29]. In an in vitro study of feldspathic ceramic crowns bonded to one-piece zirconia implants, composite resin cements yielded higher fracture loads than temporary cements; however, the authors did not quantify tensile retention or the effects of different cementation protocols [30]. Thus, the combined effects of crown material, resin cement type, and cementation protocol on the retention of crowns cemented to one-piece zirconia implants remain insufficiently understood.

### 1.4. Internal Cement Morphology and Micro-CT Analysis

Beyond cement type, the internal morphology of the cement layer, including film thickness and void distribution, may influence stress transfer and mechanical behavior in bonded ceramic crowns [31,32].

Micro-computed tomography (micro-CT) is an established non-destructive method for three-dimensional assessment of internal fit, cement film thickness, and voids in ceramic restorations and implant-supported crowns [31,32,33]. However, to our knowledge, no study has directly correlated micro-CT-derived cement layer characteristics with tensile pull-out forces in crowns cemented onto one-piece zirconia implants. Furthermore, the effect of an abutment-assisted apical-half cementation protocol on internal cement morphology in this specific configuration has not been previously investigated.

In a recent companion investigation, micro-CT was used to characterize the thickness of the cement layer, porosity, and excess cement for lithium disilicate crowns on zirconia one-piece implants, using the same cementation protocols [34].

Building on this morphological dataset, the present study focused on the mechanical behavior by quantifying tensile retention and evaluating its association with previously obtained micro-CT parameters.

Accordingly, this pilot in vitro study aimed to explore differences in tensile retention of monolithic zirconia and lithium disilicate crowns cemented onto zirconia one-piece implants using two contemporary resin cements, an adhesive resin cement (ARC), and a self-adhesive resin cement (SARC), under two cementation protocols: conventional and abutment-apical half. For lithium disilicate crowns, micro-CT-derived cement film thickness and porosity were analyzed to explore their potential association with tensile pull-out forces.

On an exploratory basis, the first null hypothesis was that tensile retention would not differ according to crown material, resin-cement type, and cementation protocol. The second exploratory null hypothesis was that micro-CT-derived internal cement film thickness and porosity would not be associated with tensile retention in lithium disilicate crowns.

## 2. Materials and Methods

This exploratory pilot in vitro study investigated the tensile retention of lithium disilicate and zirconia crowns cemented onto one-piece zirconia implants, using two resin cements and two cementation protocols. Lithium disilicate specimens had previously undergone micro-CT assessment of the cement layer morphology in a companion study.

The present work reports the maximum tensile pull-out force recorded on the same specimens and investigates its relationship with previously obtained micro-CT parameters.

### 2.1. Specimen Preparation and Crown Fabrication

A total of 32 identical one-piece zirconia implants (WhiteSky 7, Bredent, Senden, Germany) were assigned to 2 groups by the cementation of the crown material: lithium disilicate crowns (IPS e.max CAD, Ivoclar Vivadent, Schaan, Liechtenstein) and zirconia crowns (IPS e.max ZirCAD, Ivoclar Vivadent), with *n* = 16 per group. A single zirconia implant abutment was digitized using a laboratory scanner (Medit Identica Hybrid; Medit, Seoul, Republic of Korea). Based on this scan, a standardized maxillary first-premolar crown was designed using DentalCAD 3.2 Elefsina software (exocad GmbH, Darmstadt, Germany).

Sixteen crowns were milled from the IPS e.max computer-aided design (CAD) blocks (shade MTA1/C14) using a Programill PM7 unit with the corresponding PrograMill CAM V4 software, 2024 update (Ivoclar Vivadent, Schaan, Liechtenstein). After milling, the crowns were crystallized in a Programat EP5000 furnace according to the manufacturer’s recommendations, characterized, glazed, and hand-polished with rotary rubber polishers and diamond polishing paste to produce a high-gloss surface.

**Figure 1:**
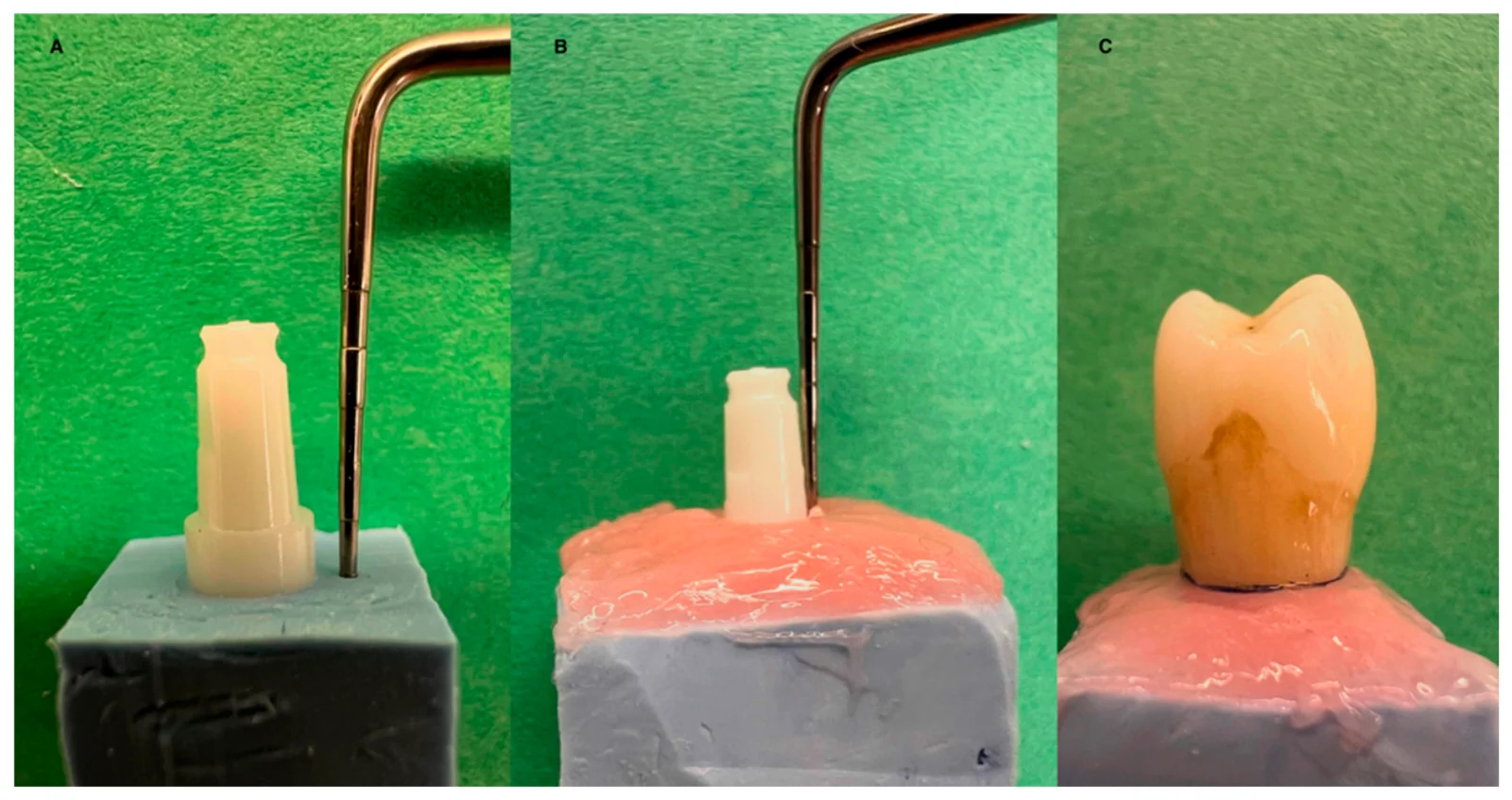
Specimen preparation and embedding procedure. (**A**) One-piece zirconia implant embedded in a silicone block to simulate supporting bone. (**B**) Application of silicone gingival mask to reproduce soft-tissue conditions and limit cement access. (**C**) Final crown–implant assembly prior to tensile testing.

Monolithic zirconia crowns were fabricated from the IPS e.max ZirCAD MTA1 disks using the CAD described above. Sixteen crowns were fabricated. The design was performed in Exocad (Exocad GmbH, Darmstadt, Germany), and milling was carried out on a ROLAND DWX-52DCi unit using hyperDENT Compact NEW V9.4 CAM software (FOLLOW-ME! Technology GmbH, Munich, Germany). The minimum crown thickness was standardized at 1.5 mm, and the virtual cement gap was set to 50 microns across the entire internal surface of all crowns (Figure 1).

### 2.2. Cement Materials and Experimental Groups

#### Two Dual-Cure Resin Cements Were Investigated

Panavia V5 (Kuraray Noritake Dental, Tokyo, Japan), an adhesive resin cement (ARC), was used with separate ceramic primers.

SpeedCem Plus (Ivoclar Vivadent, Schaan, Liechtenstein) is a self-adhesive resin cement (SARC) containing an integrated primer.

Within each crown material group (*n* = 16), implant–crown assemblies were further allocated according to cement type (Panavia V5 or SpeedCem Plus; *n* = 8 per cement), and each cement group was subsequently subdivided into two cementation protocol subgroups (AH or A-AH; *n* = 4 per subgroup).

Apical Half (AH) cement was applied only to the apical half of the crown’s internal surface.

Abutment-Apical Half (A-AH) cement was applied to the apical half with an indexing step on an abutment analog prior to final seating.

### 2.3. Cementation Procedures

All implants were embedded in silicone putty (Aquasil Ultra+ Soft Putty, Dentsply Sirona, Bensheim, Germany) such that the simulated mucosal level coincided with the transition between the endosseous and transmucosal portions of the implant (approximately 2 mm above the implant shoulder). A silicone gingival mask (UFI Gel P, VOCO, Cuxhaven, Germany) was placed around each implant to mimic the soft tissue and limit access for cement removal.

Prior to cementation, crown intaglio surface pretreatment was performed according to the crown material and cement system. In the lithium disilicate/Panavia V5 group, the crown intaglio surfaces were etched with 9% hydrofluoric acid for 20 s, rinsed with water for 30 s, air-dried, and treated with Clearfil Ceramic Primer Plus according to the manufacturer’s instructions. In the lithium disilicate/SpeedCem Plus group, the crown intaglio surfaces were conditioned with Monobond Etch and Prime according to the manufacturer’s instructions, thoroughly rinsed with water, and dried with oil-free air. All zirconia crowns were airborne-particle abraded with 50 µm Al_2_O_3_ and cleaned with oil-free air. In the zirconia/Panavia V5 group, Clearfil Ceramic Primer Plus was applied to the intaglio surfaces according to the manufacturer’s instructions. In contrast, no additional chemical surface treatment was performed on the zirconia/SpeedCem Plus group prior to cementation (Table 1).

To minimize variability in cement layer thickness attributable to cement quantity, the amount of cement used per crown was gravimetrically standardized. Before cement application, each crown was weighed using a precision balance (Sartorius, VWR International, Lutterworth, UK). The cement was then dispensed into the crown, and the crown was reweighed. This process was repeated until the cement mass per specimen reached 0.04 g.

### 2.4. Cement Application Protocols

#### 2.4.1. AH Protocol

A standardized cement volume was applied over the apical half of the crown intaglio surface. The crown was seated directly on the implant abutment.

#### 2.4.2. A-AH Protocol

The cement was applied in the same manner as in the AH protocol. The crown was first placed on a separate abutment analog (index) to pre-distribute the cement. After lifting the crown off the analog, it was immediately seated on the definitive implant.

In all groups, the crowns were pressed into place by a single operator using consistent manual pressure to approximate the clinical seating. Excess cement was removed with a carbon fiber scaler once tack curing was achieved, followed by additional cleaning after final polymerization, as required.

### 2.5. Micro-CT Dataset from Previous Investigation

The lithium disilicate specimens included in the present study were the same as those previously analyzed using micro-CT in a companion study by Azizi Bunjaku et al. [34]. The present study used previously published micro-CT variables from that study, including cement thickness and porosity, for comparative analysis with the newly generated tensile retention outcomes. No new micro-CT scans, segmentations, or measurements were performed for the present manuscript.

### 2.6. Retention Testing

Following completion of micro-CT, all implant–crown assemblies were subjected to a tensile pull-off test using a universal testing machine (Hegewald & Peschke, Nossen, Germany).

**Figure 2:**
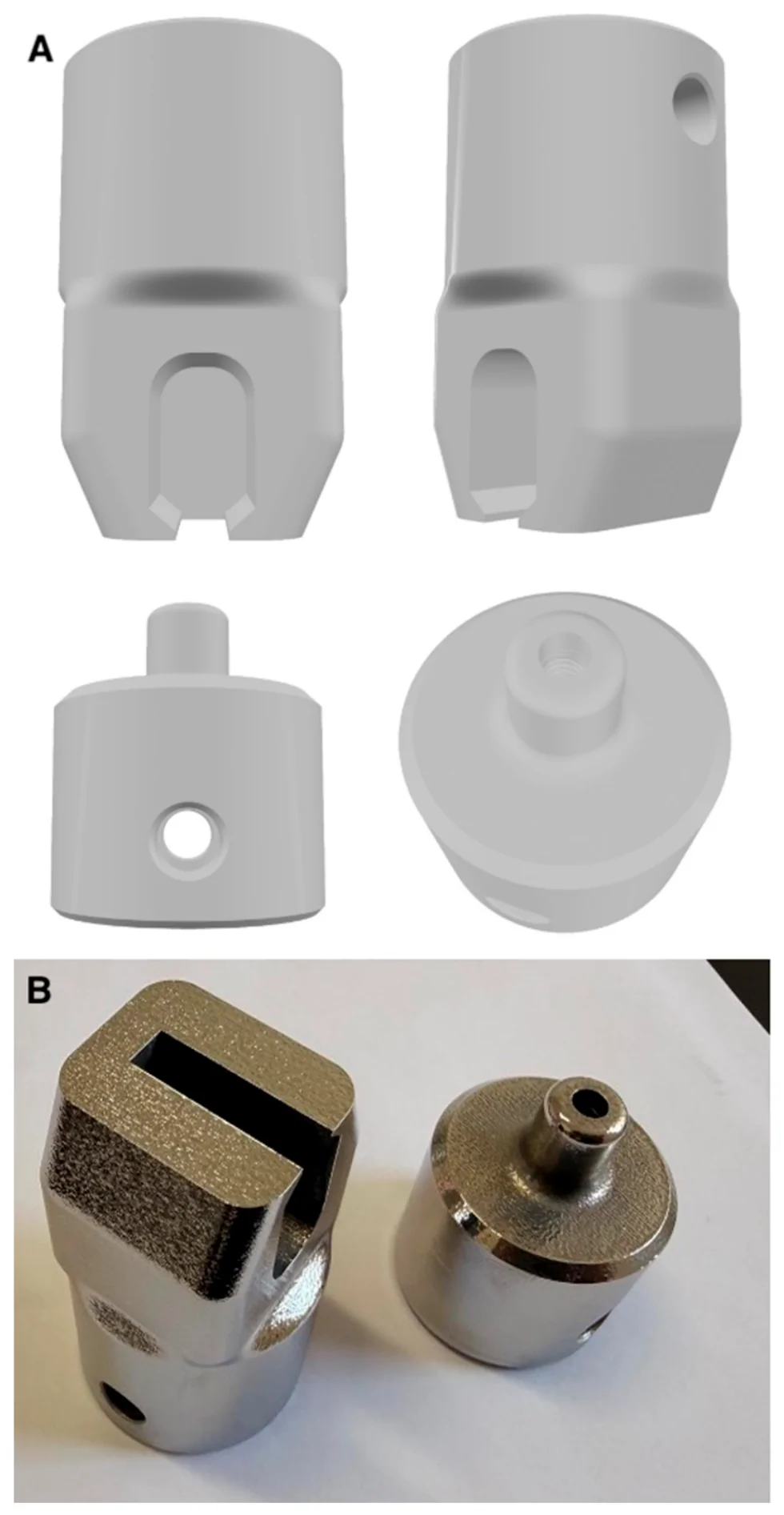
Design and fabrication of custom cobalt–chromium fixtures used for tensile testing. (**A**) CAD renderings of the upper gripping component and lower implant holder shown in multiple views. (**B**) Corresponding machined fixtures used in the experimental setup. The geometry ensured stable implant fixation and symmetric crown engagement, enabling coaxial load transfer during axial pull-out testing.

Two custom-made metallic fixtures were designed using CAD software and fabricated from a cobalt–chromium alloy to enable controlled axial tensile testing. The upper component was designed to provide symmetric engagement of the crown. In contrast, the lower holder incorporated a central threaded channel and a lateral fixation screw to ensure stable positioning of the implant (Figure 2).

The geometry of the fixtures was specifically designed to align the long axis of the implant with the loading axis of the universal testing machine, thereby minimizing off-axis loading and rotational moments.

**Figure 3:**
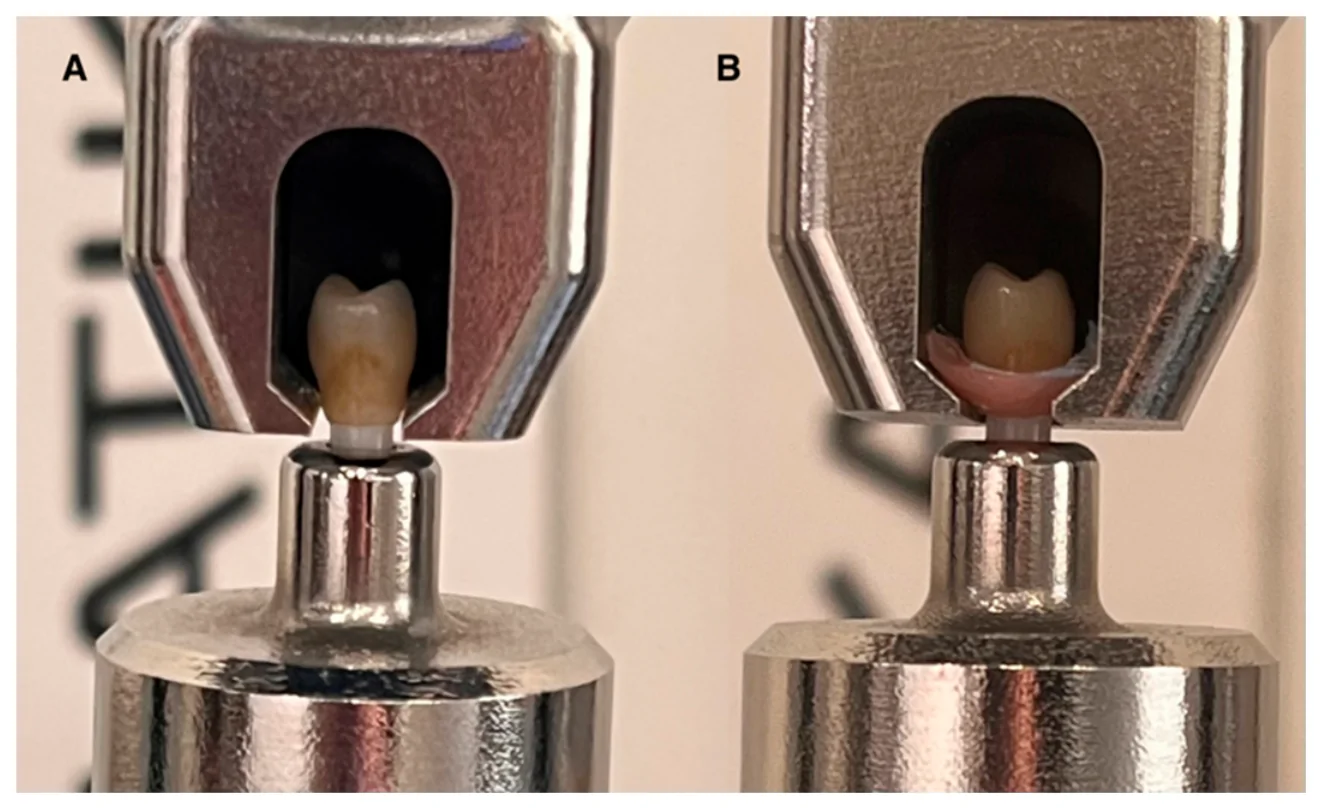
Experimental setup for axial tensile pull-out testing. (**A**) Representative crown–implant assembly mounted in the universal testing machine without a protective interlayer (illustrative configuration). (**B**) Final configuration for all mechanical testing, including a compliant protective interlayer between the crown and gripping device to minimize stress concentration during loading. In all specimens, the implant long axis was aligned coaxially with the loading axis of the testing machine to ensure pure axial tensile loading.

During testing, the lower fixture secured the implant at the base of the universal testing machine, and the upper gripping component was mounted on the crosshead. A compliant protective silicone interlayer was placed between the crown and gripping device to reduce localized stress concentrations during load application. The specimens were subjected to monotonic axial pull-off loading at a crosshead speed of 0.5 mm/min until the first mechanical failure occurred. The primary outcome was the maximum recorded force (N) at that first observed failure, irrespective of the mode of failure, so that every specimen contributed one value. For each specimen, the mode of the first failure was recorded and classified as crown debonding (recorded in the source data as “Crown decementation”), cohesive crown fracture, or implant fracture. Crown debonding was additionally analyzed as a secondary, cause-specific outcome, in which specimens that fractured before debonding were right-censored at their maximum recorded force; censoring was applied only in that secondary analysis (Section 2.7, Figure 3).

Failure mode at the first observed mechanical event was reviewed by I. P. and N. D., both blinded to experimental group allocation.

### 2.7. Statistical Analysis

Statistical analyses were performed using the SAS 9.4M9 (TS1M9) software (SAS Institute Inc., Cary, NC, USA); the raw-data reanalysis reported in this revision was carried out independently in Python 3 (pandas, SciPy and lifelines) on the same specimen-level dataset, which is reproduced in full in Supplementary Table S1. Descriptive statistics, including sample size, mean, standard deviation (SD), median and range, were calculated for the maximum recorded force, cement thickness and porosity, with the data stratified by crown material, cement type and cementation protocol.

*Primary outcome.* The primary outcome was the maximum recorded force at the first observed mechanical failure, irrespective of the mode of that failure. Because every specimen failed, all 32 observations are complete and no censoring is involved. The three balanced marginal contrasts (crown material, resin cement and cementation protocol, each 16 versus 16 specimens) were compared using the exact two-sided Mann–Whitney U test, and the common-language effect size A (the probability that a randomly drawn specimen from one group exceeds one from the other) is reported alongside each comparison.

*Secondary cause-specific outcome.* Crown debonding was analyzed separately as a cause-specific secondary outcome. In that analysis only, the applied force at which the event occurred was used as the duration scale in Cox proportional hazards regression, and the specimens whose first failure was a crown or implant fracture were treated as right-censored at their maximum recorded force. Because the duration scale is applied force rather than time, a hazard ratio greater than 1 indicates a higher instantaneous occurrence of the specified failure at a given applied force, conventionally interpreted as lower resistance to that failure under this loading protocol. Kaplan–Meier curves were used for visualization of the same cause-specific outcome.

*Censoring assumption.* The censoring in the secondary analysis cannot be presumed to be non-informative. Fracture and debonding are competing mechanical responses of the same crown–cement–implant complex, and the specimens that fractured did so at forces within the range over which debonding was also observed; a specimen that fractures may therefore differ systematically in its debonding resistance from one that does not. Standard right-censoring treats the censored specimens as if they would have debonded according to the same hazard as the specimens that remained at risk, which is not verifiable in this dataset and cannot be tested with 27 events. The debonding-only estimates are therefore reported as an exploratory description of one failure mode and not as the study’s primary result. Two sensitivity analyses were prespecified for this revision: the rank-based comparison of the maximum recorded force in all 32 specimens (no censoring), and a Cox model fitted to a composite endpoint in which any first failure counted as an event (again no censoring).

*Model specification and stability.* Cox models were restricted to the three experimental factors entered as main effects, with zirconia, SpeedCem Plus and the AH protocol as reference categories. No subgroup-specific or interaction models were fitted to the whole sample, because each of the eight subgroups contained four specimens and such models estimate more parameters than the data can support. The proportional hazards assumption was assessed for every covariate by the rank correlation between the raw Schoenfeld residuals and the rank of the applied force at which events occurred; with 27 to 32 events, this diagnostic is low-powered, so a non-significant result does not establish proportionality.

*Cement morphology.* Micro-CT-derived cement thickness and porosity exist only for the 16 lithium disilicate specimens; the corresponding values for the zirconia specimens are unavailable, not zero, because the radiopacity of zirconia prevented reliable segmentation of the cement layer. Differences in cement thickness and porosity between cements and protocols were analyzed using factorial analysis of variance (ANOVA) within the lithium disilicate specimens. The association of thickness and porosity with debonding was examined using univariable Cox models within the same 16 specimens only. All 16 thickness observations lie between 253.29 and 254.96 µm, a total span of 1.67 µm, so a per-10 µm contrast lies far outside the observed data and yields an uninterpretable hazard ratio; cement thickness was therefore scaled per 0.1 µm, which lies inside the observed range, and porosity per percentage point. Rescaling a covariate changes only the units in which the hazard ratio is expressed and leaves the model fit, the *p*-value, and the proportional hazards diagnostic unchanged. No multivariable and no interaction model of cement morphology was fitted, because the lithium disilicate subset contains 12 debonding events, the thickness covariate varies over only 1.67 µm, and that variation is itself associated with cementation protocol; a model estimating three parameters from 12 events under such restricted and confounded variation cannot yield stable estimates. As exploratory context only, rank correlations between the micro-CT parameters and the maximum recorded force were computed across all 16 lithium disilicate specimens irrespective of failure mode. No morphology result is extrapolated to the zirconia specimens.

*Interpretation of p-values.* Sample size estimation was performed in the companion study using G*Power version 3.1.9.7 (Heinrich Heine University Düsseldorf, Düsseldorf, Germany) and was based on micro-CT outcomes; therefore, the present retention analysis was not independently powered, and no claim of adequate power for any retention comparison is made. Because each experimental subgroup contained four specimens, all inferential analyses were considered exploratory, and no correction for multiplicity was applied. *p*-values are reported to describe patterns in this pilot dataset and must not be interpreted as confirmatory evidence. A non-significant result is reported as absence of evidence of an association and is never interpreted as evidence that no association exists. The nominal level of statistical significance was set at α = 0.05 for these exploratory analyses.

## 3. Results

### 3.1. Maximum Recorded Force at First Failure and Failure Modes

The maximum recorded force at the first observed failure was available for all 32 specimens and is summarized by subgroup, together with the mode of that failure, in Table 2. Failure occurred by crown debonding in 27 specimens, by cohesive crown fracture in 4 and by implant fracture in 1; all four crown fractures occurred in lithium disilicate subgroups and the single implant fracture in a zirconia subgroup. Medians and ranges are reported alongside the subgroup means because the subgroup distributions overlap substantially: the zirconia/Panavia V5/A-AH subgroup spanned 86.83 to 575.96 N and the lithium disilicate/SpeedCem Plus/AH subgroup spanned 330.47 to 746.75 N. In exact two-sided Mann–Whitney comparisons of the three balanced marginal contrasts of 16 versus 16 specimens, the maximum recorded force was lower for lithium disilicate than for zirconia crowns (medians 347.20 versus 596.05 N; U = 191, *p* = 0.017, A = 0.746) and lower with the A-AH than with the AH protocol (medians 304.24 versus 614.38 N; U = 213, *p* < 0.001, A = 0.832), whereas the comparison between the two resin cements was not statistically significant (medians 438.88 versus 550.83 N; U = 162, *p* = 0.210, A = 0.633). These comparisons are unadjusted and exploratory; the non-significant cement comparison indicates absence of evidence of a difference and not equivalence of the two cements. Specimen-level values for all 32 assemblies are given in Supplementary Table S1.

### 3.2. Secondary Cause-Specific Exploratory Analysis of Crown Debonding

**Figure 4:**
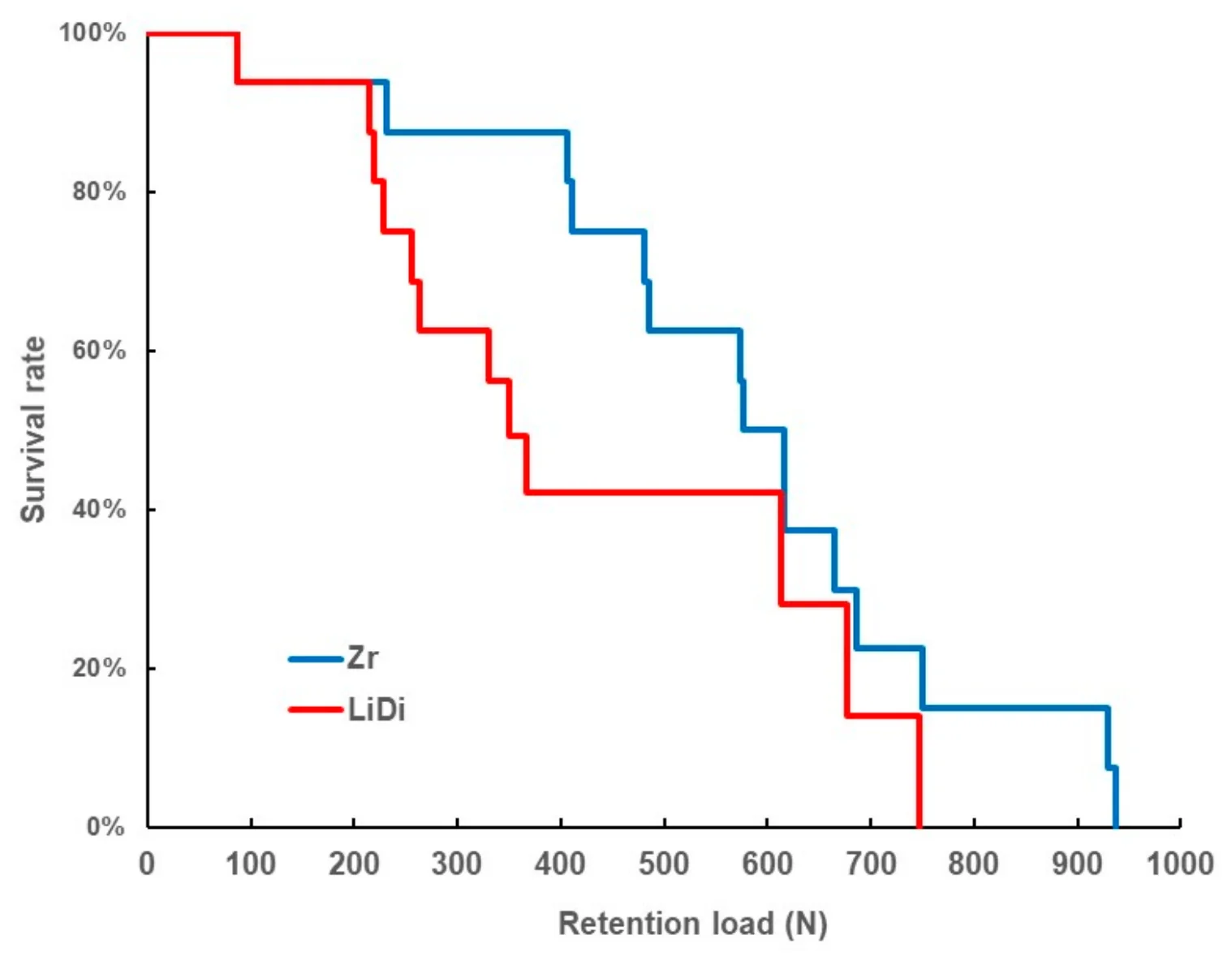
Kaplan–Meier curves. Comparison of retention load by crown materials.

**Figure 5:**
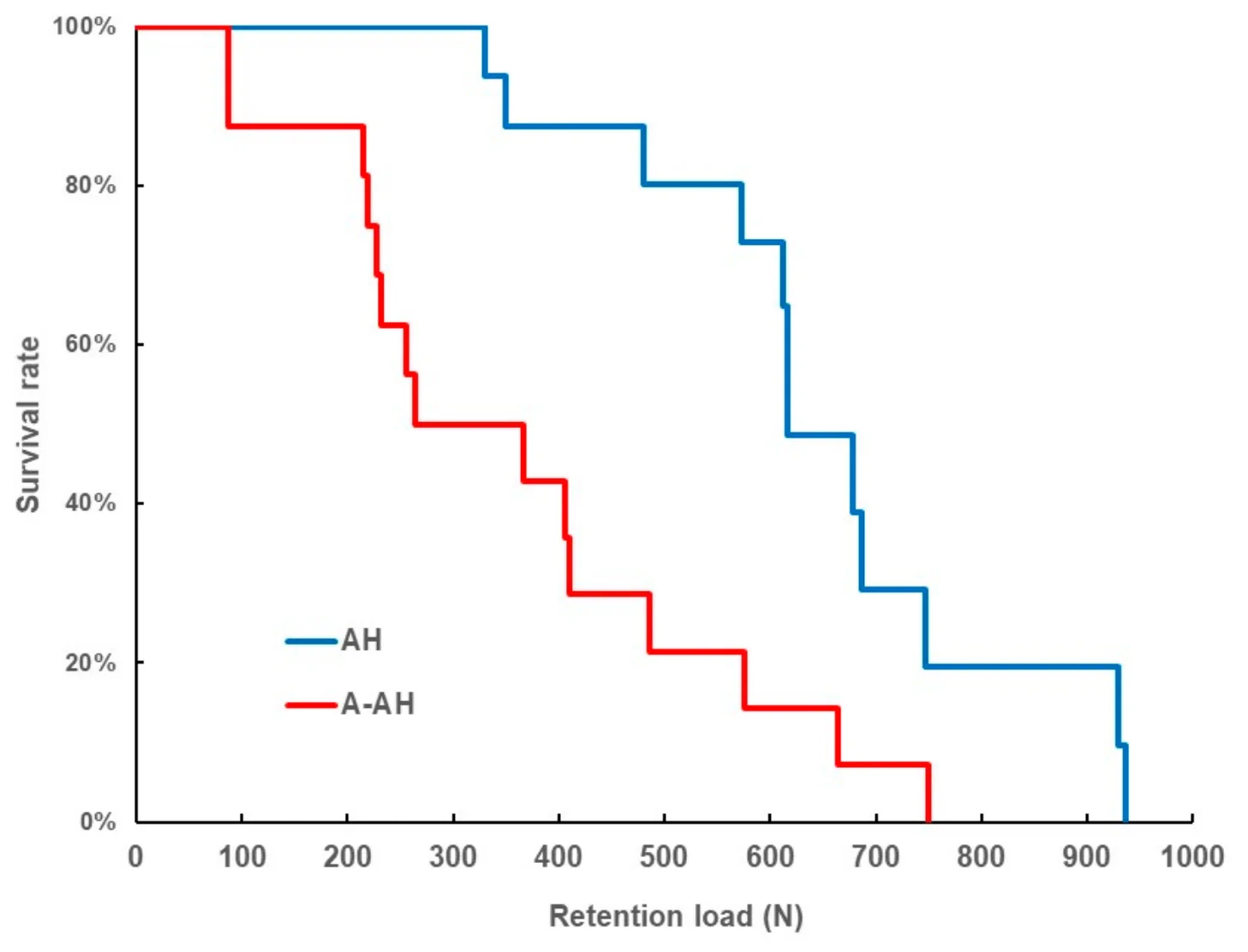
Kaplan–Meier curves. Comparison of retention load by cementation protocols.

**Figure 6:**
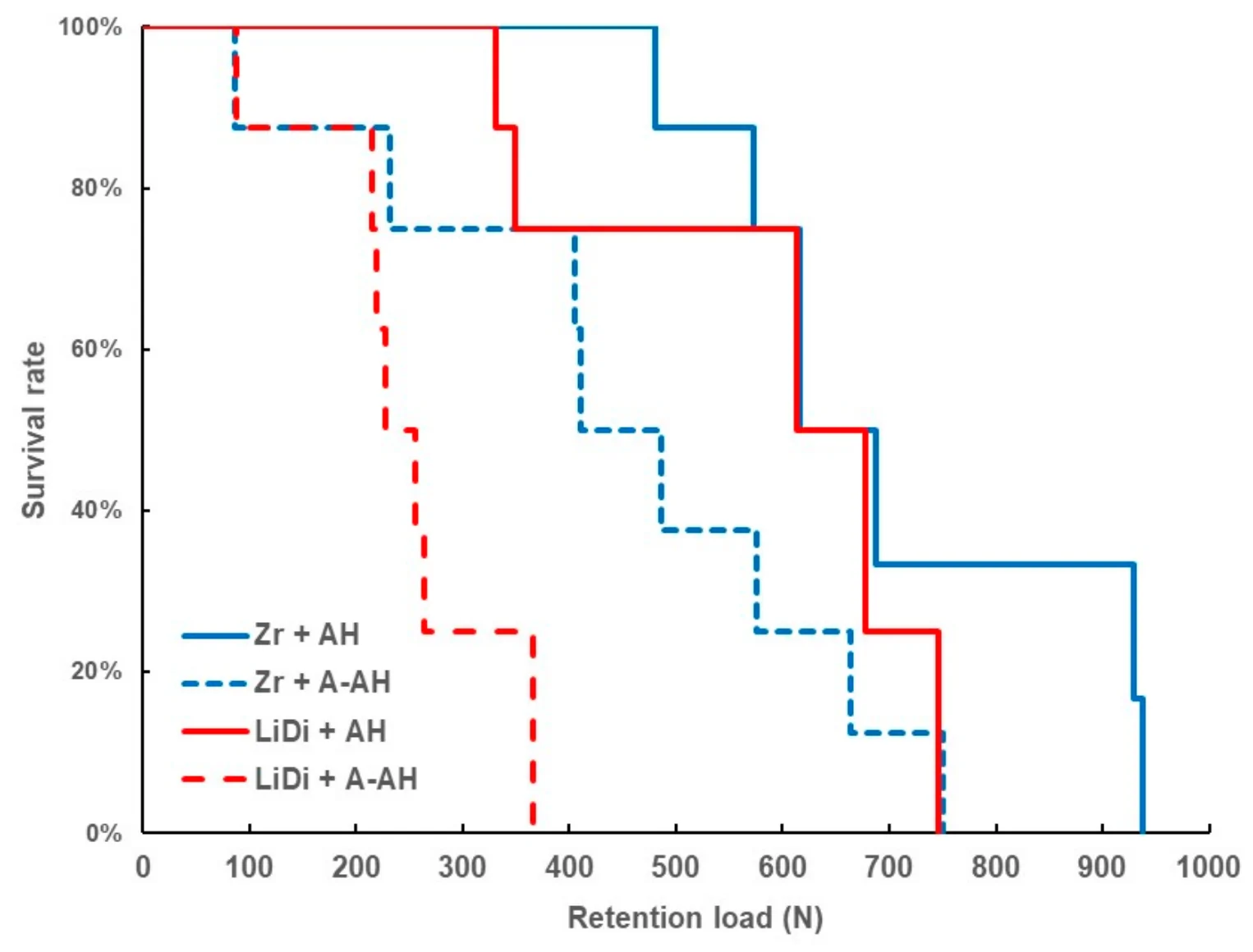
Kaplan–Meier curves. Comparison of retention load by crown materials and cementation protocols.

Crown debonding was additionally analyzed as a cause-specific secondary outcome on the applied-force scale, with the five specimens that fractured before debonding right-censored at their maximum recorded force. This analysis rests on an assumption of non-informative censoring that cannot be verified in this dataset (Section 2.7) and is therefore reported as an exploratory description of a single failure mode. With 27 observed debonding events, the model containing the three experimental factors as main effects gave hazard ratios of 3.75 (95% CI 1.40–10.01; *p* = 0.008) for lithium disilicate versus zirconia, 6.47 (2.39–17.53; *p* < 0.001) for the A-AH versus the AH protocol and 1.82 (0.76–4.39; *p* = 0.181) for Panavia V5 versus SpeedCem Plus (Table 3). Because the applied force rather than elapsed time is the duration scale, a hazard ratio above 1 indicates a higher instantaneous occurrence of debonding at a given applied force, conventionally interpreted as lower resistance to that failure under this loading protocol. Kaplan–Meier curves for the same cause-specific outcome are shown in Figure 4, Figure 5 and Figure 6.

The proportional hazards assumption was assessed for each covariate by the rank correlation between the raw Schoenfeld residuals and the rank of the applied force at which events occurred. No departure was detected for any covariate (crown material rho = 0.048, *p* = 0.811; resin cement rho = −0.042, *p* = 0.834; cementation protocol rho = −0.162, *p* = 0.421). With 27 events, this diagnostic is low-powered, and the absence of a detected departure does not establish that the proportional hazards assumption holds.

### 3.3. Sensitivity Analyses

Two prespecified sensitivity analyses examined whether the direction of the observed differences depended on how the five fracture events were handled (Table 4). First, the rank-based comparisons of the maximum recorded force reported in Section 3.1 use all 32 specimens and involve no censoring. Second, the Cox model was refitted to a composite endpoint in which any first failure counted as an event at the maximum recorded force, so that nothing was censored; with 32 events the hazard ratios were 4.76 (95% CI 1.93–11.77; *p* < 0.001) for lithium disilicate versus zirconia, 6.01 (2.38–15.18; *p* < 0.001) for A-AH versus AH and 1.99 (0.89–4.44; *p* = 0.094) for Panavia V5 versus SpeedCem Plus. Rank correlations between the raw Schoenfeld residuals and the applied-force rank again detected no departure from proportional hazards (crown material rho = 0.039, *p* = 0.831; resin cement rho = −0.019, *p* = 0.918; cementation protocol rho = −0.221, *p* = 0.224), subject to the same low-power caveat. The direction of every estimate was preserved across the three analyses and the confidence intervals overlapped substantially, which indicates that the observed pattern is not an artifact of the censoring rule. This is internal consistency and not external validation: all three analyses use the same 32 recorded forces, every interval remains wide, and no comparison is adjusted for multiplicity.

### 3.4. Cement Thickness and Porosity (Lithium Disilicate Crowns Only)

**Figure 7:**
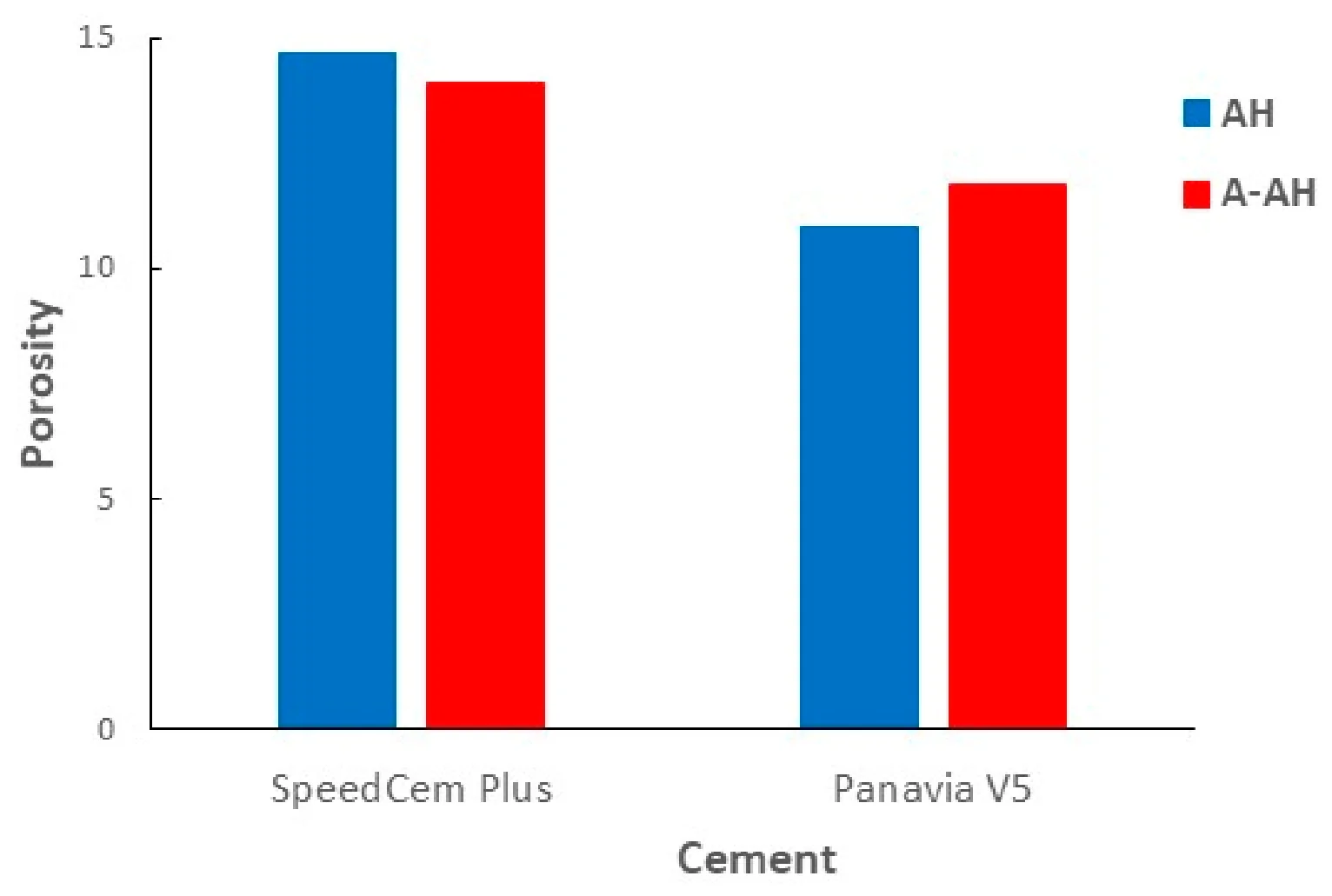
Comparison of porosity by cement and cementation protocols.

Micro-CT-derived cement thickness and porosity were available only for the 16 lithium disilicate specimens; the corresponding values for the zirconia specimens are unavailable rather than zero, because the radiopacity of zirconia prevented reliable segmentation of the cement layer. Descriptive values by subgroup are given in Table 5. All 16 thickness observations lay between 253.29 and 254.96 µm and the four subgroup means ranged from 253.92 to 254.73 µm, so the total observed variation in cement layer thickness was 1.67 µm. Within the lithium disilicate specimens, factorial ANOVA indicated a statistically significant association between cementation protocol and cement layer thickness (*p* = 0.035), with marginally higher observed thickness values under A-AH than under AH (Table 6). No statistically significant association was detected between resin cement type and thickness (*p* = 0.844), and the resin cement × protocol interaction was not statistically significant for thickness (*p* = 0.285); neither result is evidence that no such association exists. Porosity was significantly associated with resin cement type (*p* < 0.001), with a higher mean porosity observed for SpeedCem Plus than for Panavia V5 (Table 7, Figure 7), whereas no statistically significant association was detected for cementation protocol (*p* = 0.826) or for the interaction (*p* = 0.268). All six terms of the two factorial models were computed directly from the specimen-level values in Supplementary Table S1 and are reproducible from that table. Every statement in this subsection is restricted to the lithium disilicate specimens, and none of it is extrapolated to the zirconia specimens.

### 3.5. Association Between Cement Morphology and Debonding (Lithium Disilicate Crowns Only)

In the lithium disilicate subset (*n* = 16, 12 observed debonding events, 4 specimens censored at crown fracture), univariable exploratory Cox models gave a hazard ratio for debonding of 1.24 per 0.1 µm of cement thickness (95% CI 1.03–1.48; *p* = 0.024) and of 0.959 per percentage point of total porosity (0.717–1.283; *p* = 0.776) (Table 8). The thickness estimate must not be read as a definitive thickness finding. It is an unadjusted univariable association observed across a total thickness range of only 1.67 µm (253.29–254.96 µm); that range is itself associated with cementation protocol; and expressing the identical model per 10 µm, a step far outside the observed data, returns a hazard ratio of 1.51 × 10^9^ (95% CI 16.22–1.40 × 10^17^; *p* = 0.024), which shows how weakly the data constrain the slope. The estimate therefore does not demonstrate a clinically meaningful or an independent effect of cement thickness, and it cannot support a dose–response or a causal interpretation. As exploratory context only, and across all 16 lithium disilicate specimens irrespective of failure mode, the rank correlation between cement thickness and the maximum recorded force was rho = −0.527 (*p* = 0.036) and that between total porosity and the maximum recorded force was rho = 0.062 (*p* = 0.820); these correlations reinforce the restricted-range caveat rather than resolve it. Within the same subset, the corresponding univariable estimates for the experimental factors were 1.19 (0.34–4.15; *p* = 0.780) for Panavia V5 versus SpeedCem Plus and 9.31 (1.81–47.87; *p* = 0.008) for A-AH versus AH (Table 8). The previously reported multivariable model containing cement thickness, porosity and their interaction has been removed: it estimated three parameters from 12 events, and the thickness covariate varies over only 1.67 µm and is associated with cementation protocol, so no stable adjusted estimate can be obtained from these data. The non-significant porosity result is not evidence that porosity is unrelated to retention; with 12 events, the confidence interval still includes effects that would be mechanically meaningful.

Table 2 summarizes the primary outcome across all experimental subgroups together with the observed failure modes. It is the baseline reference for the raw means, SDs, medians and ranges against which the subsequent exploratory models are compared.

To describe the probabilistic nature of crown debonding across the applied-force range, the secondary cause-specific outcome is displayed as Kaplan–Meier curves in Figure 4, Figure 5 and Figure 6. These curves show the cumulative probability of remaining bonded as the applied force increases rather than relying on a single mean value, and they refer to debonding only; the five specimens whose first failure was a fracture are censored in them.

Figure 4 shows separation in the observed cause-specific debonding probability between zirconia and lithium disilicate crowns, consistent with the hazard ratio of 3.75 (95% CI 1.40–10.01; *p* = 0.008) estimated in the secondary exploratory Cox model.

Figure 5 shifts the focus to the cementation protocol and shows separation between the AH and A-AH protocols (hazard ratio 6.47, 95% CI 2.39–17.53; *p* < 0.001), illustrating lower observed resistance to debonding with the abutment-assisted apical-half strategy.

Figure 6 presents the combined debonding patterns observed across crown materials and cementation protocols. This figure supports a descriptive comparison among all experimental groups; no interaction model was fitted to the whole sample, because each subgroup contains four specimens.

Following these observed mechanical patterns, the analysis turns to the micro-CT-derived internal cement layer measurements to explore whether morphological variation might be associated with retention.

**Figure 8:**
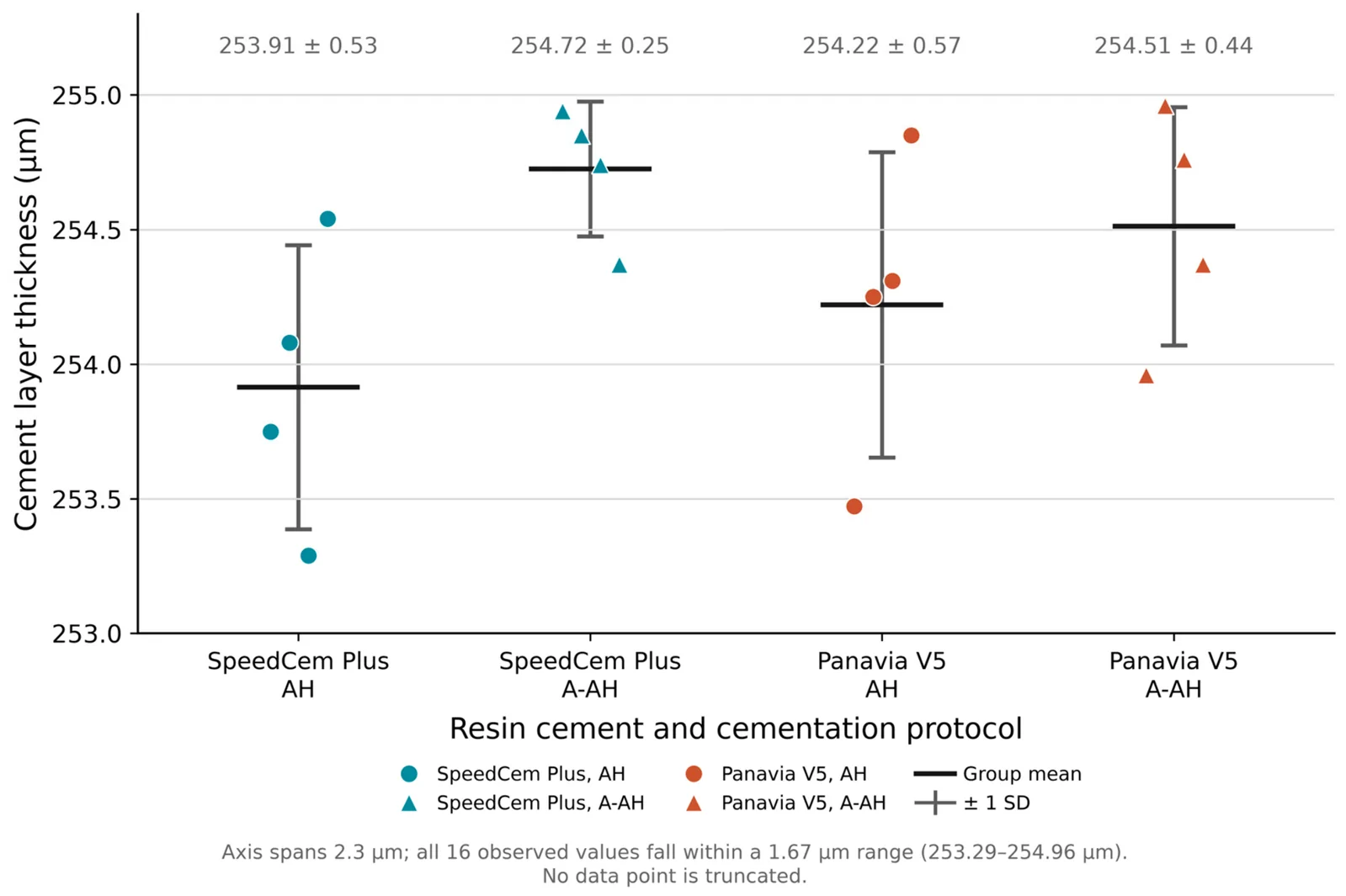
Cement thickness in lithium-disilicate specimens by resin cement and protocol. Points represent individual specimens; horizontal bars and error bars indicate group means and SD. Color denotes the resin cement, and marker shape denotes the cementation protocol. All 16 observations lie between 253.29 and 254.96 µm; the vertical axis is restricted to that region so that no value is truncated.

Table 5 gives the descriptive cement thickness and porosity values for the lithium disilicate specimens. Table 6 and Figure 8 summarize the analysis of variance for cement thickness, while Table 7 and Figure 7 present the corresponding porosity findings.

Figure 8 shows every individual cement thickness measurement in the four lithium disilicate subgroups, together with the subgroup mean and standard deviation. It complements the ANOVA results in Table 6 by illustrating the association with cementation protocol (*p* = 0.035). The vertical scale is deliberately restricted to the region actually occupied by the data, because all 16 values lie between 253.29 and 254.96 µm; the visible separation between subgroups therefore corresponds to differences of well under one micrometer.

Figure 7 presents the porosity measurements across the lithium disilicate groups. This visualization complements the thickness data and the ANOVA results in Table 7, indicating a statistically significant association between porosity and resin cement type (*p* < 0.001).

Table 8 summarizes the univariable exploratory Cox models fitted within the lithium disilicate subset. An unadjusted association with debonding was observed for cement thickness expressed per 0.1 µm (*p* = 0.024), across a total observed range of 1.67 µm that is itself associated with cementation protocol, so it does not demonstrate a clinically meaningful or independent thickness effect. No statistically significant association was detected for total porosity (*p* = 0.776); with 12 debonding events and a wide confidence interval, this is an absence of evidence, and it is not evidence that no association exists.

## 4. Discussion

This pilot in vitro study evaluated the tensile retention of lithium disilicate and monolithic zirconia crowns cemented directly onto zirconia one-piece implants and examined associations with crown material, resin cement type, and cementation protocol. The primary outcome was the maximum recorded force at the first observed mechanical failure, irrespective of the mode of that failure. The relationship between micro-CT-derived cement layer characteristics and retention was also explored, for the lithium disilicate specimens only. The preliminary results suggested associations of crown material and cementation protocol with the observed retention pattern, whereas no statistically significant difference between the two resin cements was detected. Within the lithium disilicate specimens, cement layer thickness varied over a total range of only 1.67 µm; an unadjusted univariable association with debonding was observed on that restricted scale, but it is confounded with cementation protocol and does not demonstrate a clinically meaningful or an independent thickness effect, while no statistically significant association with total porosity was detected. Each non-significant result reflects absence of evidence in a pilot dataset of four specimens per subgroup and must not be read as evidence that no association exists.

### 4.1. The Influence of Crown Material and Retention

From a materials science perspective, the retention behavior observed in the present study may be explained by the interaction between crown substrate composition, resin cement chemistry, and seating protocol. Lithium disilicate is a silica-based glass-ceramic that can be micromechanically conditioned by hydrofluoric acid etching and chemically coupled to resin cement through silane-mediated bonding [1,3,4]. In contrast, zirconia is a polycrystalline oxide ceramic that does not form a silica-rich etched surface and therefore relies primarily on airborne-particle abrasion and phosphate-monomer-mediated chemical bonding, particularly through MDP-containing systems [1,5,6,7]. Panavia V5 is an adhesive resin cement used with a separate ceramic primer [22,23], whereas SpeedCem Plus is a self-adhesive resin cement with an integrated primer [23,29]. Although differences in cement chemistry and application protocol may influence interfacial bonding, film formation, and porosity [31,32,35], the preliminary results of this study suggest that crown material and cementation protocol may have contributed more strongly to the observed retention patterns than the specific resin cement system under monotonic tensile loading.

In this pilot dataset, crown material was associated with a statistically significant difference in the maximum recorded force at first failure, and zirconia crowns showed higher observed pull-out loads than lithium disilicate crowns. This pattern may be related to the distinct mechanical behavior of these ceramics. Yttria-stabilized zirconia exhibits a higher elastic modulus, flexural strength, and fracture toughness than lithium disilicate, largely owing to transformation toughening mechanisms that inhibit crack initiation and propagation [1,5]. Finite element analysis has demonstrated that stiffer crown materials concentrate more stress within their own structure during loading, exhibiting less interfacial deformation compared with more flexible materials [36]. This reduced tendency for deformation at the crown–cement–abutment interface may help preserve the mechanical engagement between the crown and abutment under axial tensile loading. Under monotonic tensile loading, retention is influenced not only by adhesive bond strength but also by the elastic response of the crown–cement–abutment complex, which may therefore have contributed to the observed retention pattern. That all four cohesive crown fractures occurred in lithium disilicate specimens is consistent with this interpretation, although with four fractures it cannot be tested.

Although lithium disilicate is capable of strong adhesive bonding following hydrofluoric acid etching and silanization, it exhibits lower fracture toughness and greater sensitivity to surface defects [1,3]. Therefore, the stress concentrations or microcracks at the intaglio surface may become critical at lower tensile loads, thereby contributing to earlier failure. While some in vitro studies have reported higher pull-off forces for zirconia-based ceramics than for lithium disilicate crowns on implant abutments, others have indicated that lithium disilicate can achieve comparable retention depending on the surface pretreatment and cementation protocol [29,37].

Therefore, the higher retention observed for zirconia crowns in this pilot dataset may be partly explained by material-related mechanical factors; however, the study design does not permit a definitive mechanistic conclusion.

Nevertheless, the retention outcomes remain dependent on the specific material combination, abutment configuration, and luting protocol employed.

### 4.2. Cementation Protocol and Retention

The conventional apical half (AH) cementation protocol showed higher observed pull-out forces than the abutment-apical half (A-AH) protocol. This finding is consistent with previous reports showing that conventional single-step cementation yields higher retention than techniques involving pre-distribution of cement on a separate abutment before final seating [38].

One possible explanation is that direct single-step seating may contain more of the luting agent within the interface before polymerization and may thereby increase mechanical engagement at the crown-abutment interface. This interpretation remains tentative and requires confirmation.

### 4.3. Cement Thickness and Porosity

A previous micro-CT investigation of the same specimens reported statistically significant differences in cement film thickness between cementation protocols and in porosity between resin cement types [34]. In the lithium-disilicate specimens, cement thickness ranged from 253.29 to 254.96 µm at the specimen level, with subgroup means from 253.92 to 254.73 µm (Table 5). These values exceeded the 50 µm virtual cement space defined during CAD. Because the entire observed range spans only 1.67 µm and is associated with the cementation protocol, the univariable association between thickness and debonding reported in Section 3.5 cannot be interpreted as a clinically meaningful or independent thickness effect, and no association between porosity and debonding was detected; with 12 debonding events, the latter is an absence of evidence and not evidence that no association exists. The higher observed retention with the AH protocol may therefore be related to procedural factors, such as the seating dynamics and containment of the luting agent during direct placement, rather than to absolute cement film thickness. This tentative interpretation aligns with previous findings indicating that cement thickness may be more relevant within substantially thinner ranges.

Mehl et al. reported a statistically significant association between cement film thickness and retention only within a narrow 15–50 µm range, with no relevant changes observed beyond this interval [28]. Accordingly, the cement thickness values observed in the present study may have exceeded the range within which further increases are associated with measurable changes in tensile retention.

### 4.4. Resin Cement Type

Within this pilot dataset, no statistically significant difference in tensile retention between the two resin cements was detected (*p* = 0.210 for the primary outcome). The adhesive resin cement (Panavia V5) and the self-adhesive resin cement (SpeedCem Plus) showed broadly comparable observed initial retention values under monotonic tensile loading, but with 16 specimens per cement, the confidence interval around the corresponding hazard ratio (0.76–4.39) still admits a substantial difference in either direction, so this is an absence of evidence rather than evidence of equivalence. This preliminary pattern is consistent with the findings from implant-related in vitro studies suggesting that, once effective chemical adhesion to zirconia or titanium bases is established with contemporary MDP-containing resin cements, variations between specific resin products tend to produce smaller changes in pull-out or fracture strength than factors such as abutment height, abutment/Ti-base geometry, cement space, or surface pretreatment [23,29].

A systematic review by Shadid investigated factors influencing the bond strength between CAD-CAM implant-supported ceramic restorations and Ti-bases, including Ti-base height and geometry, various surface pretreatments, type of luting cement, and luting space thickness [29]. The authors concluded that the Ti-base design, abutment height, luting space thickness, and surface pretreatment consistently influenced bond strength and retention. In contrast, differences among contemporary MDP-containing resin cements were often secondary when similar bonding protocols were used [29].

The cement layer morphology was characterized using micro-CT in a previous companion study on the same specimens [34]; these data are summarized here to contextualize the present retention findings.

The companion micro-CT study reported statistically significant associations of resin cement type and cementation protocol with cement layer morphology in the lithium disilicate specimens [34]. Importantly, the micro-CT and tensile retention analyses were performed on the same specimen cohort, allowing a direct exploratory comparison between cement layer morphology and retention outcomes within those specimens. Despite these reported morphological differences, the present univariable Cox models detected no statistically significant association between debonding and total porosity, and the association observed for cement thickness (1.24 per 0.1 µm, 95% CI 1.03–1.48) is an unadjusted estimate across a 1.67 µm observed range that is itself associated with cementation protocol. Given 12 debonding events, a confidence interval of 0.717–1.283 per percentage point for porosity and an essentially one-dimensional thickness covariate, the data cannot resolve whether cement morphology influences initial retention; this is not evidence that the observed morphological differences failed to translate into differences in initial retention, nor evidence that thickness itself drives it.

The micro-CT dataset showed significant differences in cement thickness between cementation protocols and in porosity between cement types, within the lithium disilicate specimens. On the authoritative specimen-level data, the A-AH protocol was associated with marginally higher observed cement thickness than the AH protocol, and SpeedCem Plus showed higher observed porosity than Panavia V5 (Table 5, Table 6 and Table 7). The absolute thickness difference is small: every value lies between 253.29 and 254.96 µm. These observations are consistent with previous micro-CT findings suggesting that the cement type and application procedure influence the cement film thickness and void formation [31,32,35]. Total porosity was not significantly associated with debonding in the univariable Cox models, and the univariable thickness association is confounded with cementation protocol over a 1.67 µm range. The present data therefore do not resolve whether micro-CT-detectable differences in cement morphology affect the force required to debond the crowns, and they do not establish that they do not. Any suggestion that further increases in cement thickness beyond a certain level may not proportionally enhance friction-based mechanical retention between crown and abutment therefore remains a hypothesis for adequately powered testing.

Comparable discrepancies between the virtual cement space generated in CAD software and the cement film or internal gap observed have been reported for other CAD/CAM restorations. Dauti et al. showed that adjusting the spacer parameter from 50 to 80 µm for polymer-infiltrated ceramic network (PICN) crowns did not significantly change the marginal or internal fit, with measured gaps remaining well above the nominal spacer values [32].

These deviations are attributed, among others, to the limited reproducibility of die spacer layers, manufacturing and scanning tolerances, preparation or abutment geometry, particularly the convergence angle and internal line angles of axial walls, seating dynamics that govern excess cement expression, and the occurrence of premature internal contacts [32,34]. Taken together, these findings provide a mechanistic rationale for analyzing the relationship between cement thickness and retention in the present study.

Porosity within the resin cement layer of these specimens had already been characterized in the companion study and was therefore not re-measured for the present study. The previously obtained porosity values were included in the exploratory association analysis with retention, for the lithium disilicate specimens only. Under monotonic pull-out loading, the influence of porosity may be less apparent than under long-term fatigue or cyclic loading [39]. Nevertheless, micro-CT investigations have demonstrated that voids and pores in the luting layer can be segmented and quantified and that porosities located at the margin and open to the oral environment represent structural discontinuities and potential pathways for fluid and bacterial ingress [32,40]. In resin cements, polymerization shrinkage generates internal stresses that can promote interfacial gap formation and crack propagation, which have been linked to microleakage, discoloration, secondary caries, fatigue fractures, and restoration failure over time [41,42]. Thus, although no measurable reduction in immediate tensile retention was detected at the porosity levels present in these specimens, porosity remains a potential risk factor for the long-term mechanical and interfacial stability of cement-retained restorations.

In contrast to several previous investigations on titanium-based or two-piece abutments, the present study focused on the direct cementation of zirconia one-piece implants and directly quantified cement morphology using micro-CT.

Prior in vitro studies and a recent systematic review have shown that resin-based luting agents generally provide higher retention than provisional or glass-ionomer-based cements for implant-supported crowns [23,29,43]. Several studies have reported comparable retention among contemporary resin cements under standardized conditions [23,24,29,37]. Within this context, the present findings are consistent with that literature but do not extend it: in this pilot dataset, no difference between the two resin cements was detected, and the data are too sparse to establish that moderate differences in cement morphology leave initial tensile retention unchanged under monotonic loading.

### 4.5. Clinical Implications

The findings of this pilot study apply specifically to one-piece zirconia implants, which inherently preclude screw retention, and frequently present with submucosal crown margins. In this context, the cementation strategy may be relevant to balancing mechanical stability and biological risk [17]. Excess cement was not measured in the present analysis. In the companion micro-CT investigation of the same specimens, the AH protocol was associated with a greater volume of excess cement than the A-AH protocol [34]; the specimen-level excess-cement volumes reproduced in Supplementary Table S1 come from that investigation and are descriptive context only, with no inferential analysis performed here. Excess cement is a risk indicator for peri-implant mucositis and peri-implantitis and is particularly problematic when crown margins are located submucosally, limiting access for cement removal [19,20,21].

The A-AH protocol was associated with less excess cement in the companion investigation [34], whereas in the present pilot dataset it showed lower observed retention. Taken together, and treating the excess-cement evidence as exploratory context from the companion study rather than as an outcome of the present analysis, these preliminary findings suggest that cementation protocol selection may warrant consideration alongside cement type in one-piece zirconia implant restorations [23,30,44,45,46].

### 4.6. Limitations

This pilot study was limited by its in vitro design and the use of monotonic tensile loading, which does not fully replicate the complex mechanical and environmental conditions of the oral cavity. Environmental factors such as moisture exposure, water sorption, hydrolytic degradation, and thermal fluctuations were not evaluated, and thermomechanical aging procedures, including water storage, thermocycling, and fatigue loading, were not performed. Micro-CT analysis was restricted to the lithium disilicate specimens because accurate characterization of the cement layer was not feasible in the zirconia groups owing to the high radiopacity of zirconia; the corresponding zirconia values are therefore unavailable rather than zero, and every morphology result applies to the lithium disilicate specimens alone. Most importantly, each experimental subgroup contained only four specimens, and sample-size estimation had been based on the companion micro-CT outcomes rather than on the present retention outcome, so no retention comparison in this study is adequately powered. The large standard deviations in several subgroups indicate substantial within-group variability, which limits precision and makes the findings sensitive to random variation and specimen-level inconsistencies. For that reason, no subgroup-specific and no interaction Cox model was fitted to the whole sample, and the multivariable model of cement morphology reported previously has been removed: with four specimens per subgroup and 12 to 32 events overall, such models estimate more parameters than the data support. A further limitation specific to the micro-CT parameters is that cement layer thickness varies over only 1.67 µm across all 16 lithium disilicate specimens (253.29–254.96 µm) and that this variation is associated with cementation protocol, so no adjusted thickness estimate can be separated from the protocol effect and the univariable thickness association must not be read as a dose–response or causal relationship. The secondary debonding analysis additionally requires an assumption of non-informative censoring that cannot be verified here, since fracture and debonding are competing responses of the same assembly (Section 2.7); the sensitivity analyses in Table 4 show only that the direction of the estimates is stable across censoring rules, which is internal consistency and not external statistical validation. No analysis was corrected for multiplicity, all confidence intervals are wide, and every non-significant result is reported as absence of evidence rather than as evidence of no association. The inferential analyses and *p*-values should therefore be considered exploratory, and the observed group differences interpreted as preliminary and hypothesis-generating rather than confirmatory. Larger, independently powered studies incorporating aging protocols and alternative imaging approaches are needed to confirm or refute the observed patterns and clarify their clinical relevance.

## 5. Conclusions

Within the limitations of this pilot in vitro study, the preliminary findings suggest that crown material and cementation protocol may be associated with the maximum force sustained before the first mechanical failure of crowns cemented to one-piece zirconia implants. Zirconia crowns showed higher observed pull-out loads than lithium disilicate crowns, and the conventional apical-half cementation protocol showed higher observed loads than the abutment-assisted apical-half protocol. No statistically significant difference between the two resin cements was detected. Within the lithium disilicate specimens, cementation protocol was associated with differences in cement layer thickness and resin cement type with differences in porosity; no statistically significant association between total porosity and retention was detected, and the univariable association observed for cement thickness spans a total range of only 1.67 µm that is confounded with cementation protocol and therefore does not demonstrate a clinically meaningful or an independent thickness effect. Because each subgroup contained four specimens, all confidence intervals are wide, and no comparison was corrected for multiplicity, each of these non-significant results indicates absence of evidence rather than evidence of no association. These results should be regarded as preliminary and hypothesis-generating and require confirmation in larger, independently powered studies.

## Figures and Tables

**Table 1:** Reported material properties of Panavia V5 and SpeedCEM Plus, including chemical composition, mechanical and physical characteristics. Adapted from our previously published companion study [34].

Property	Panavia V5	SpeedCEM Plus
Classification	Adhesive dual-cure resin cement	Self-adhesive dual-cure resin cement
Manufacturer	Kuraray Noritake	Ivoclar Vivadent
Functional monomers	MDP, Bis-GMA, TEGDMA, hydrophobic aromatic dimethacrylate	UDMA, TEGDMA, PEGDMA, DDDMA, MDP
Filler loading	61 wt% (38 vol%)	75 wt% (Base)/69.8 wt% (Catalyst)
Flexural strength (MPa)	127	≥60
Flexural modulus (GPa)	6.3	4.81
Compressive strength (MPa)	310	Not reported
Film thickness (µm)	12	33.8 ± 5.3
Water sorption (µg/mm^3^)	21	30.74 ± 2.4
Working time (23 °C)	2 min	120–180 s
Filler composition	Silanated barium glass filler, colloidal silica, silanated fluoroaluminosilicate glass filler	Barium glass, silica, ytterbium trifluoride

Data obtained from manufacturer technical documentation and the published literature.

**Table 2:** Maximum recorded force at the first observed mechanical failure and mode of that failure, by experimental subgroup (*n* = 4 per subgroup, 32 specimens in total).

Crown Material	Resin Cement	Protocol	*n*	Debonding	Crown Fracture	Implant Fracture	Mean (N)	SD (N)	Median (N)	Min–Max (N)
Zr	SpeedCem Plus	AH	4	3	0	1	638.9	33.3	626.34	616.13–686.79
Zr	SpeedCem Plus	A-AH	4	4	0	0	577.7	156.8	574.91	410.50–750.39
Zr	Panavia V5	AH	4	4	0	0	729.9	237.7	751.17	480.11–937.16
Zr	Panavia V5	A-AH	4	4	0	0	324.9	212.1	318.42	86.83–575.96
LiDi	SpeedCem Plus	AH	4	3	1	0	554.7	191.9	570.78	330.47–746.75
LiDi	SpeedCem Plus	A-AH	4	4	0	0	275.0	64.6	259.47	214.63–366.38
LiDi	Panavia V5	AH	4	2	2	0	503.3	118.6	525.44	349.71–612.63
LiDi	Panavia V5	A-AH	4	3	1	0	219.7	105.1	223.23	87.63–344.70
Total	—	—	32	27	4	1	—	—	—	—

Note: Zr = zirconia; LiDi = lithium disilicate; AH = conventional apical-half cementation protocol; A-AH = abutment-assisted apical-half cementation protocol; SD = standard deviation. The mode of the first observed failure is tabulated for every specimen, so the three failure-mode columns sum to n in each row and to 32 overall. Crown debonding corresponds to the source records beginning “Crown decementation”; crown fracture denotes cohesive fracture within the crown. Means, SDs, medians and ranges are calculated from the maximum recorded force irrespective of failure mode, so all four specimens contribute to every subgroup statistic.

**Table 3:** Secondary cause-specific exploratory Cox proportional hazards model for crown debonding on the applied-force scale (*n* = 32; 27 debonding events; 5 specimens right-censored at fracture). Main effects only.

Covariate (Reference Category)	Hazard Ratio	95% CI	*p*	Schoenfeld Rho	Schoenfeld *p*
Lithium disilicate (vs. zirconia)	3.75	1.40–10.01	0.008	0.048	0.811
Panavia V5 (vs. SpeedCem Plus)	1.82	0.76–4.39	0.181	−0.042	0.834
A-AH protocol (vs. AH)	6.47	2.39–17.53	<0.001	−0.162	0.421

Note: the duration scale is applied force, not time, so a hazard ratio greater than 1 indicates a higher instantaneous occurrence of debonding at a given applied force, conventionally interpreted as lower resistance to that failure under this loading protocol. Schoenfeld columns give the rank correlation between the raw Schoenfeld residuals and the rank of the applied force at which events occurred; with 27 events, this diagnostic is low-powered. The censoring is not demonstrably non-informative (Section 2.7), and the non-significant cement estimate indicates absence of evidence rather than evidence of no association.

**Table 4:** Sensitivity of the observed associations to the handling of the five specimens whose first failure was a fracture.

Analysis	Contrast (Reference)	Estimate (95% CI)	*p*
Primary: maximum recorded force at first failure, all 32 specimens, no censoring (exact Mann–Whitney)	Lithium disilicate vs. zirconia	Medians 347.20 vs. 596.05 N; U = 191; A = 0.746	0.017
	Panavia V5 vs. SpeedCem Plus	Medians 438.88 vs. 550.83 N; U = 162; A = 0.633	0.210
	A-AH vs. AH	Medians 304.24 vs. 614.38 N; U = 213; A = 0.832	<0.001
Secondary: cause-specific Cox, crown debonding only (27 events, 5 censored)	Lithium disilicate vs. zirconia	HR 3.75 (1.40–10.01)	0.008
	Panavia V5 vs. SpeedCem Plus	HR 1.82 (0.76–4.39)	0.181
	A-AH vs. AH	HR 6.47 (2.39–17.53)	<0.001
Sensitivity: Cox, composite any-first-failure endpoint (32 events, none censored)	Lithium disilicate vs. zirconia	HR 4.76 (1.93–11.77)	<0.001
	Panavia V5 vs. SpeedCem Plus	HR 1.99 (0.89–4.44)	0.094
	A-AH vs. AH	HR 6.01 (2.38–15.18)	<0.001

Note: HR = hazard ratio; A = common-language effect size. All three analyses use the same 32 recorded forces. Proportional hazards diagnostics for the composite model were rho = 0.039, *p* = 0.831 (crown material), rho = −0.019, *p* = 0.918 (resin cement) and rho = −0.221, *p* = 0.224 (cementation protocol). Agreement between the three analyses is internal consistency only; it is not external statistical validation, no interval is narrow, and no comparison is adjusted for multiplicity.

**Table 5:** Micro-CT-derived cement layer thickness and total porosity of the lithium disilicate specimens, by resin cement and cementation protocol (*n* = 4 per subgroup, 16 specimens). Equivalent measurements are unavailable for the zirconia specimens.

Resin Cement	Cementation Protocol	*n*	Cement Thickness, Mean ± SD (µm)	Thickness Min–Max (µm)	Total Porosity, Mean ± SD (%)	Porosity Min–Max (%)
SpeedCem Plus	AH	4	253.915 ± 0.528	253.29–254.54	14.650 ± 0.971	13.37–15.44
SpeedCem Plus	A-AH	4	254.725 ± 0.250	254.37–254.94	14.014 ± 1.032	12.82–15.18
Panavia V5	AH	4	254.221 ± 0.567	253.473–254.85	10.885 ± 2.183	8.86–13.98
Panavia V5	A-AH	4	254.513 ± 0.442	253.96–254.96	11.826 ± 0.784	11.06–12.91

Note: values are reproduced from the companion micro-CT investigation of the same specimens [34]; no new scans or measurements were performed. All 16 specimen-level thickness values lie between 253.29 and 254.96 µm, a total range of 1.67 µm, and are listed in Supplementary Table S1. Between-group comparisons of these descriptive values gave *p* = 0.959 for thickness by resin cement type, *p* = 0.038 for thickness by protocol, *p* = 0.002 for porosity by resin cement type and *p* = 0.959 for porosity by protocol (exact Mann–Whitney, 8 versus 8 specimens, unadjusted and exploratory). Equivalent measurements are unavailable, not zero, for the 16 zirconia specimens.

**Table 6:** Analysis of variance (ANOVA) of cement thickness (lithium disilicate specimens only, *n* = 16).

Source	df	Sum of Squares	Mean Square	F	*p*
Resin cement	1	0.0087	0.0087	0.041	0.844
Cementation protocol	1	1.2139	1.2139	5.657	0.035
Resin cement × cementation protocol	1	0.2686	0.2686	1.252	0.285
Error	12	2.5748	0.2146	—	—

Note: revised two-way factorial ANOVA of cement layer thickness within the 16 lithium disilicate specimens (error df = 12), computed on the specimen-level values listed in Supplementary Table S1. Every term in this table, including the sums of squares and mean squares, is reproduced exactly from Supplementary Table S1; because the design is balanced with four specimens per cell, Type I, Type II and Type III sums of squares are identical. All 16 thickness observations lie within a 1.67 µm range, so a statistically significant protocol term corresponds to a very small absolute difference. Non-significant terms indicate absence of evidence of an association, not evidence of no association.

**Table 7:** Analysis of variance (ANOVA) of total porosity (lithium disilicate specimens only, *n* = 16).

Source	df	Sum of Squares	Mean Square	F	*p*
Resin cement	1	35.438	35.438	19.187	0.0009
Cementation protocol	1	0.093	0.093	0.051	0.826
Resin cement × cementation protocol	1	2.489	2.489	1.347	0.268
Error	12	22.164	1.847	—	—

Note: revised two-way factorial ANOVA of total porosity within the 16 lithium disilicate specimens (error df = 12), computed on the specimen-level values listed in Supplementary Table S1. Every term in this table, including the sums of squares and mean squares, is reproduced exactly from Supplementary Table S1; the balanced design with four specimens per cell makes Type I, Type II and Type III sums of squares identical. Non-significant terms indicate absence of evidence of an association, not evidence of no association.

**Table 8:** Univariable exploratory Cox models for crown debonding within the lithium disilicate specimens (*n* = 16, 12 events, 4 censored at crown fracture).

Covariate	Hazard Ratio (95% CI)	*p*	Schoenfeld *p*
Panavia V5 vs. SpeedCem Plus	1.19 (0.34–4.15)	0.780	0.912
A-AH protocol vs. AH	9.31 (1.81–47.87)	0.008	0.547
Cement thickness, per 0.1 µm	1.24 (1.03–1.48)	0.024	0.286
Total porosity, per percentage point	0.959 (0.717–1.283)	0.776	0.561

Note: each row is a separate univariable model fitted within the 16 lithium disilicate specimens only; no multivariable and no interaction model was fitted, because the subset contains 12 debonding events. Cement thickness is expressed per 0.1 µm because all 16 observations lie within a 1.67 µm range (253.29–254.96 µm); the same model expressed per 10 µm gives a hazard ratio of 1.51 × 10^9^ (95% CI 16.22–1.40 × 10^17^) with an identical *p*-value, which is numerically uninformative. The thickness estimate is unadjusted, is confounded with cementation protocol, and does not demonstrate a clinically meaningful or an independent thickness effect. It cannot support a dose–response or causal interpretation. The non-significant result for total porosity indicates absence of evidence of an association and is not evidence that no association exists. No estimate in this table applies to the zirconia specimens.

## Data Availability

The original contributions presented in this study are included in the article/Supplementary Materials. Further inquiries can be directed to the corresponding author.

## Supplementary Materials

The following supporting information can be downloaded at: https://www.mdpi.com/article/10.3390/ma19163518/s1, Table S1: Specimen-level dataset for all 32 implant–crown assemblies.

## Abbreviations

The following abbreviations are used in this manuscript:

3Y-TZP	3 mol% yttria-stabilized tetragonal zirconia polycrystal
micro-CT/micro CT	micro-computed tomography
UTM	universal testing machine
ARC	adhesive resin cement
SARC	self-adhesive resin cement
AH	apical half
A-AH	abutment-assisted apical half
CAD	computer-aided design
CAM	computer-aided manufacturing
PICN	polymer-infiltrated ceramic network
Ti base/Ti-base/titanium base	titanium base
MDP	methacryloyloxydecyl dihydrogen phosphate functional monomer
