# Supplementary material for: Tensile Retention of Lithium Disilicate and Zirconia Crowns Cemented to One-Piece Zirconia Implants: A Pilot In Vitro Study of Cementation Protocol, Resin Cement, and Micro-CT Cement Morphology
Source: Materials (Basel). 2026 Aug 19;19(16):3518. doi: 10.3390/ma19163518 (PMC13514237; doi:10.3390/ma19163518)
Supplement: Supplementary file 1 [file materials-19-03518-s001.zip › materials-4354143-supplementary.pdf]

Supplementary Table S1 reports the complete specimen-level dataset underlying every analysis in this article. Each of the 32 implant–crown assemblies contributes one row.

Table S1. Specimen-level dataset for all 32 implant–crown assemblies.

Specimen	Crown material	Resin cement	Protocol	Force at first failure (N)	Failure mode	Cement thickness (µm)	Total porosity (%)	Excess cement (mm³)
ZS1	Zirconia	SpeedCem Plus	AH	686.79	Crown debonding	—	—	0.905
ZS2	Zirconia	SpeedCem Plus	AH	616.13	Crown debonding	—	—	1.238
ZS3	Zirconia	SpeedCem Plus	AH	616.34	Crown debonding	—	—	0.817
ZS4	Zirconia	SpeedCem Plus	AH	636.33	Implant fracture	—	—	1.287
ZS5	Zirconia	SpeedCem Plus	A-AH	750.39	Crown debonding	—	—	0.143
ZS6	Zirconia	SpeedCem Plus	A-AH	410.50	Crown debonding	—	—	0.266
ZS7	Zirconia	SpeedCem Plus	A-AH	664.30	Crown debonding	—	—	0.296
ZS8	Zirconia	SpeedCem Plus	A-AH	485.52	Crown debonding	—	—	0.005
ZP1	Zirconia	Panavia V5	AH	937.16	Crown debonding	—	—	1.811
ZP2	Zirconia	Panavia V5	AH	929.09	Crown debonding	—	—	1.164
ZP3	Zirconia	Panavia V5	AH	480.11	Crown debonding	—	—	0.809
ZP4	Zirconia	Panavia V5	AH	573.25	Crown debonding	—	—	0.875
ZP5	Zirconia	Panavia V5	A-AH	405.66	Crown debonding	—	—	0.128
ZP6	Zirconia	Panavia V5	A-AH	86.83	Crown debonding	—	—	0.182
ZP7	Zirconia	Panavia V5	A-AH	575.96	Crown debonding	—	—	0.094
ZP8	Zirconia	Panavia V5	A-AH	231.18	Crown debonding	—	—	0.131
LS1	Lithium disilicate	SpeedCem Plus	AH	330.47	Crown debonding	253.75	15.370	0.134
LS2	Lithium disilicate	SpeedCem Plus	AH	677.61	Crown debonding	254,08	13.373	0.148
LS3	Lithium disilicate	SpeedCem Plus	AH	746.75	Crown debonding	253,29	15.440	0.238
LS4	Lithium disilicate	SpeedCem Plus	AH	463.95	Crown fracture	254,54	14.417	0.138

Specimen	Crown material	Resin cement	Protocol	Force at first failure (N)	Failure mode	Cement thickness (μm)	Total porosity (%)	Excess cement (mm³)
LS5	Lithium disilicate	SpeedCem Plus	A-AH	263.79	Crown debonding	254,94	12.817	0.018
LS6	Lithium disilicate	SpeedCem Plus	A-AH	366.38	Crown debonding	254,85	13.582	0.023
LS7	Lithium disilicate	SpeedCem Plus	A-AH	255.15	Crown debonding	254,74	15.183	0.007
LS8	Lithium disilicate	SpeedCem Plus	A-AH	214.63	Crown debonding	254,37	14.474	0.005
LP1	Lithium disilicate	Panavia V5	AH	472.11	Crown fracture	253,473	8.864	0.025
LP2	Lithium disilicate	Panavia V5	AH	578.77	Crown fracture	254.25	13.984	0.041
LP3	Lithium disilicate	Panavia V5	AH	612.63	Crown debonding	254,31	10.458	0.026
LP4	Lithium disilicate	Panavia V5	AH	349.71	Crown debonding	254,85	10.233	0.018
LP5	Lithium disilicate	Panavia V5	A-AH	344.70	Crown fracture	253,96	11.784	0.010
LP6	Lithium disilicate	Panavia V5	A-AH	218.67	Crown debonding	254,96	12.910	0.017
LP7	Lithium disilicate	Panavia V5	A-AH	227.79	Crown debonding	254,76	11.555	0.025
LP8	Lithium disilicate	Panavia V5	A-AH	87.63	Crown debonding	254,37	11.056	0.015

Note: specimen identifiers follow the source dataset (Z = zirconia crown, L = lithium disilicate crown; S = SpeedCem Plus, P = Panavia V5). The maximum force is the force at the first observed mechanical failure, irrespective of its mode, and is the primary outcome of this study; all 32 values are distinct. "Crown debonding" corresponds to the source records beginning "Crown decementation", and "Crown fracture" to cohesive failure within the crown. Cement thickness and total porosity were measured by micro-CT in the companion study [34] and exist only for the 16 lithium disilicate specimens. Excess cement volumes are reproduced from the companion study for descriptive context only and were not analyzed inferentially here.
